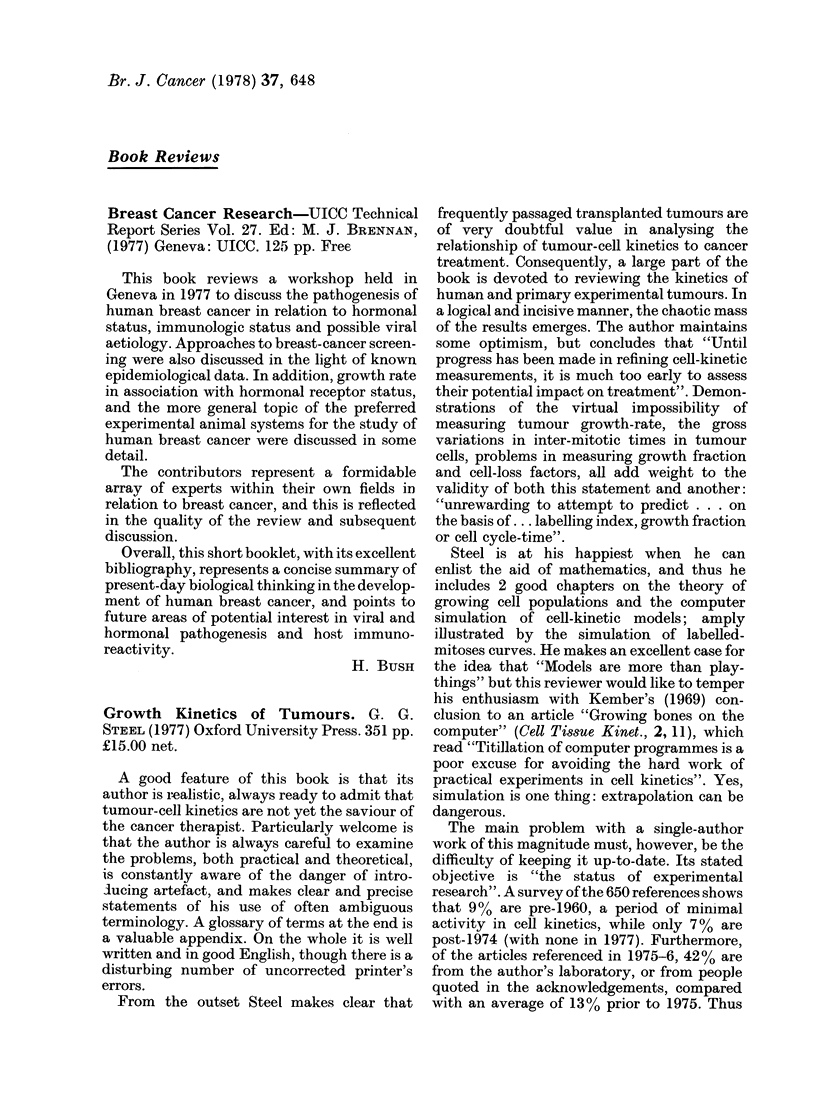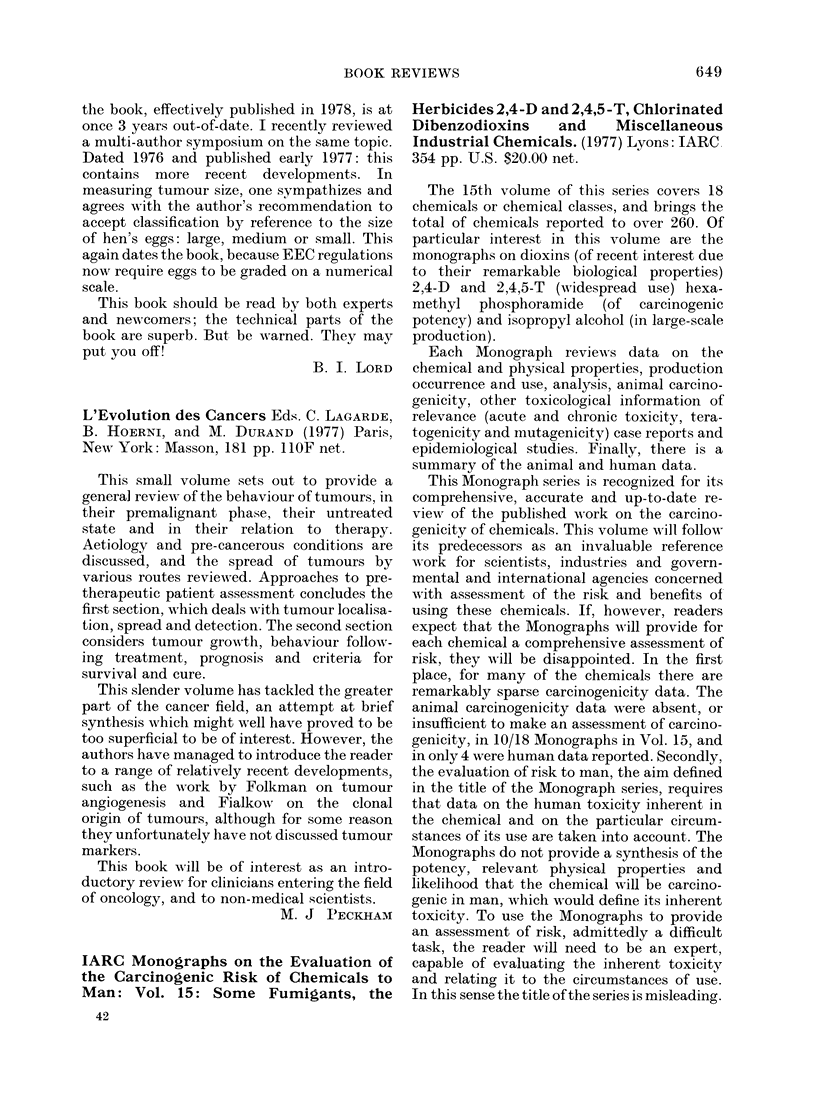# Growth Kinetics of Tumours

**Published:** 1978-04

**Authors:** B. I. Lord


**Growth Kinetics of Tumours.** G. G. Steel (1977) Oxford University Press. 351 pp. £15.00 net.

A good feature of this book is that its author is realistic, always ready to admit that tumour-cell kinetics are not yet the saviour of the cancer therapist. Particularly welcome is that the author is always careful to examine the problems, both practical and theoretical, is constantly aware of the danger of introducing artefact, and makes clear and precise statements of his use of often ambiguous terminology. A glossary of terms at the end is a valuable appendix. On the whole it is well written and in good English, though there is a disturbing number of uncorrected printer's errors.

From the outset Steel makes clear that frequently passaged transplanted tumours are of very doubtful value in analysing the relationship of tumour-cell kinetics to cancer treatment. Consequently, a large part of the book is devoted to reviewing the kinetics of human and primary experimental tumours. In a logical and incisive manner, the chaotic mass of the results emerges. The author maintains some optimism, but concludes that "Until progress has been made in refining cell-kinetic measurements, it is much too early to assess their potential impact on treatment". Demonstrations of the virtual impossibility of measuring tumour growth-rate, the gross variations in inter-mitotic times in tumour cells, problems in measuring growth fraction and cell-loss factors, all add weight to the validity of both this statement and another: "unrewarding to attempt to predict . . . on the basis of . . . labelling index, growth fraction or cell cycle-time".

Steel is at his happiest when he can enlist the aid of mathematics, and thus he includes 2 good chapters on the theory of growing cell populations and the computer simulation of cell-kinetic models; amply illustrated by the simulation of labelled-mitoses curves. He makes an excellent case for the idea that "Models are more than playthings" but this reviewer would like to temper his enthusiasm with Kember's (1969) conclusion to an article "Growing bones on the computer" (*Cell Tissue Kinet.*, **2**, 11), which read "Titillation of computer programmes is a poor excuse for avoiding the hard work of practical experiments in cell kinetics". Yes, simulation is one thing: extrapolation can be dangerous.

The main problem with a single-author work of this magnitude must, however, be the difficulty of keeping it up-to-date. Its stated objective is "the status of experimental research". A survey of the 650 references shows that 9% are pre-1960, a period of minimal activity in cell kinetics, while only 7% are post-1974 (with none in 1977). Furthermore, of the articles referenced in 1975–6, 42% are from the author's laboratory, or from people quoted in the acknowledgements, compared with an average of 13% prior to 1975. Thus the book, effectively published in 1978, is at once 3 years out-of-date. I recently reviewed a multi-author symposium on the same topic. Dated 1976 and published early 1977: this contains more recent developments. In measuring tumour size, one sympathizes and agrees with the author's recommendation to accept classification by reference to the size of hen's eggs: large, medium or small. This again dates the book, because EEC regulations now require eggs to be graded on a numerical scale.

This book should be read by both experts and newcomers; the technical parts of the book are superb. But be warned. They may put you off!

B. I. LORD